# Tripeptide-Assisted Gold Nanocluster Formation for Fe^3+^ and Cu^2+^ Sensing

**DOI:** 10.3390/molecules29112416

**Published:** 2024-05-21

**Authors:** Jonghae Youn, Peiyuan Kang, Justin Crowe, Caleb Thornsbury, Peter Kim, Zhenpeng Qin, Jiyong Lee

**Affiliations:** 1 Department of Chemistry and Biochemistry, The University of Texas at Dallas, Richardson, TX 75080, USA; jhyounnn@gmail.com (J.Y.); peter98kim@gmail.com (P.K.); 2 Department of Mechanical Engineering, The University of Texas at Dallas, Richardson, TX 75080, USA; kanglang108@hotmail.com (P.K.); zhenpeng.qin@utdallas.edu (Z.Q.); 3 Department of Chemistry and Biochemistry, The University of Texas at Tyler, Tyler, TX 75799, USA; neocrowe8@gmail.com (J.C.); cthornsbury99@gmail.com (C.T.); 4 Department of Bioengineering, The University of Texas at Dallas, Richardson, TX 75080, USA; 5 Department of Surgery, The University of Texas Southwestern Medical Center, Dallas, TX 75390, USA; 6 Center for Advanced Pain Studies, The University of Texas at Dallas, Richardson, TX 75080, USA

**Keywords:** fluorescent gold nanoclusters, tripeptides, surface ligands, metal ion sensors, aggregation-induced fluorescence quenching

## Abstract

Fluorescent gold nanoclusters (AuNCs) have shown promise as metal ion sensors. Further research into surface ligands is crucial for developing sensors that are both selective and sensitive. Here, we designed simple tripeptides to form fluorescent AuNCs, capitalizing on tyrosine’s reduction capability under alkaline conditions. We investigated tyrosine’s role in both forming AuNCs and sensing metal ions. Two tripeptides, tyrosine–cysteine–tyrosine (YCY) and serine–cysteine–tyrosine (SCY), were used to form AuNCs. YCY peptides produced AuNCs with blue and red fluorescence, while SCY peptides produced blue-emitting AuNCs. The blue fluorescence of YCY- and SCY-AuNCs was selectively quenched by Fe^3+^ and Cu^2+^, whereas red-emitting YCY-AuNC fluorescence remained stable with 13 different metal ions. The number of tyrosine residues influenced the sensor response. DLS measurements revealed different aggregation propensities in the presence of various metal ions, indicating that chelation between the peptide and target ions led to aggregation and fluorescence quenching. Highlighting the innovation of our approach, our study demonstrates the feasibility of the rational design of peptides for the formation of fluorescent AuNCs that serve as highly selective and sensitive surface ligands for metal ion sensing. This method marks an advancement over existing methods due to its dual capability in both synthesizing gold nanoclusters and detecting analytes, specifically Fe^3+^ and Cu^2+^.

## 1. Introduction

Ultrasmall gold nanoclusters (AuNCs) comprising several gold atoms have gained great interest in the field of catalysis, bioimaging, optical sensing, and biomedicine due to their unique physical and optoelectronic properties, such as ultrasmall sizes, large Stokes shifts, longer fluorescence lifetimes, photostability, biocompatibility, and the feasibility of surface functionalization [1,2,3,4,5,6,7]. When the size of an AuNC approaches the Fermi wavelength (~1 nm) of the electrons, the continuous band of energy breaks into discrete electron transition energy levels, exhibiting single-molecule-like fluorescence [8,9,10,11]. The electrons filled in 5d^10^ of the valence band are excited to 6sp^1^ of the conduction band, thereby displaying a strong fluorescence emission from the visible to the near-infrared region [10]. 

There are two common strategies, bottom–up and top–down, for the preparation of fluorescent AuNCs. The bottom–up approach is to reduce Au ions to Au atoms through the chemical, biological, optical, and electrochemical reduction of Au ions. However, due to the aggregation propensity of AuNCs, suitable surface ligands acting as stabilizers, such as small thiol molecules, polymers, peptides, and proteins, are essential for the preparation of stable AuNCs [12,13,14,15]. In the top–down approach, on the other hand, larger gold nanoparticles (~100 nm) are etched by adding excess amount of etching agents, such as polyethylenimine (PEI), dihydrolipoic acid (DHLA), or glutathione (GSH) to generate AuNCs [16,17,18].

Several synthetic methods for generating fluorescent AuNCs have been developed using ligands acting as both reducing agents and stabilizers. Such ligands include proteins, peptides, polymers, and DNA [19,20,21,22,23]. Proteins are preferred surface ligands due to the roles that several amino acids play in AuNC formation. The thiol group in cysteine residue binds to the Au precursor (Au^3+^) via strong Au-S bonds. The phenol group of tyrosine can reduce Au^3+^ into Au^0^, especially under alkaline conditions. By electron transfer, the phenol group of tyrosine becomes a semi-quinone [24,25,26,27]. Based on these previous observations, we have hypothesized that a very short peptide such as tripeptide with cystine and tyrosine in the sequence would be enough for efficient fluorescent AuNC formation. 

AuNCs have emerged as highly effective tools for developing chemical sensors due to their direct detection capabilities, high responsiveness, minimal sample requirements, and ability to monitor in real time with rapid response rates. These properties make AuNCs particularly suitable for detecting highly mutagenic metal ions like Hg^2+^, Fe^3+^, Cu^2+^, Pb^2+^, and Cr^3+^ [28,29,30,31,32,33]. Most detections utilize the mechanism of fluorescence quenching of AuNCs when exposed to specific metal ions [28,34,35]. For instance, in the detection of Hg^2+^, electron transfer typically occurs where Hg^2+^ binds strongly to Au^0^, leading to energy transfer as the excited electron of Au is transferred to Hg^2+^ [35]. In addition to Hg^2+^, fluorescence quenching in AuNCs can be induced through aggregation, where metal ions such as Fe^3+^, Cu^2+^, and Pb^2+^ form strong coordination with the ligands on adjacent AuNCs, resulting in aggregation that diminishes fluorescence [28,29,30,32]. This aggregation underscores the versatility of AuNCs, where our designed peptides enhance both the formation and sensing capabilities, utilizing the extensive research on peptide–metal chelation to inform their structure and function in these advanced applications [36,37,38,39].

This study introduced fluorescent AuNCs comprising tyrosine-containing tripeptides, tyrosine–cysteine–tyrosine (YCY) and serine–cysteine–tyrosine (SCY). A cysteine residue was employed to utilize the thiol functional group for Au-S bonding. For SCY, tyrosine residue at the N-terminus of the YCY was replaced with serine to examine the impact of the number of tyrosine residues. Blue and red fluorescence-emitting AuNCs from the YCY peptide (Blue-YCY-AuNC and Red-YCY-AuNC, respectively, hereafter) and blue fluorescence-emitting SCY-AuNC (Blue-SCY-AuNC hereafter) were obtained. Subsequently, we investigated the metal ion-sensing ability of these AuNCs. We found that Fe^3+^ and Cu^2+^ can effectively suppress the fluorescence emission of AuNCs, while other metal ions at the same concentration did not cause any quenching. We propose a plausible mechanism in which chelation between the peptide on the AuNC surface and the target ions results in aggregation and causes fluorescence quenching.

## 2. Results and Discussion

### 2.1. Preparation of AuNCs

**Scheme 1:**
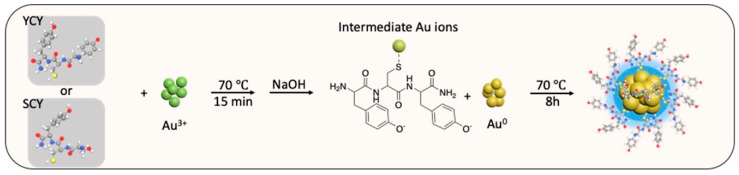
Schematic illustration of the preparation of fluorescent AuNCs via tyrosine-assisted reduction. Intermediate Au ions with an SCY peptide is not shown for simplicity.

**Figure 1:**
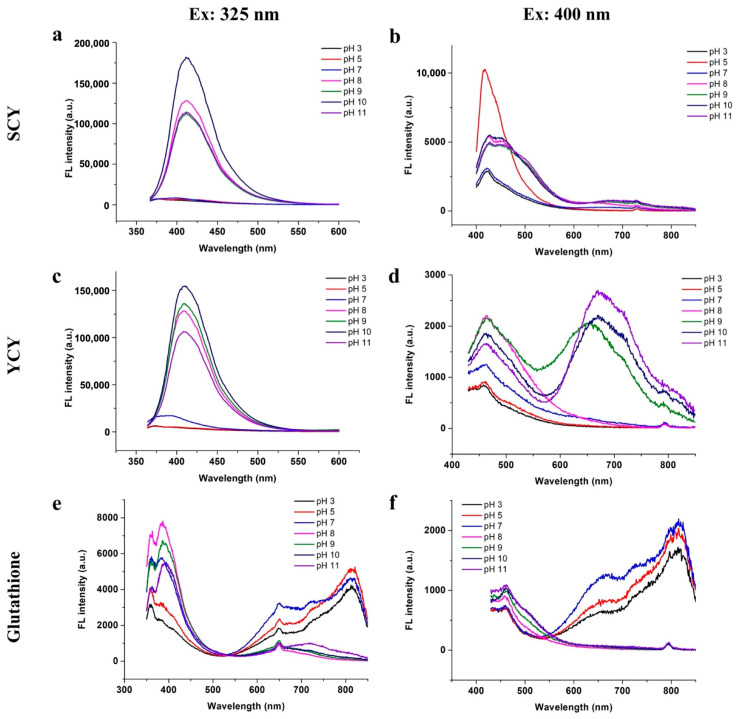
Fluorescent emission spectra of the crude AuNC solutions prepared by mixing with different peptides at the different pH conditions with 325 nm and 400 nm excitations. (**a**,**b**): SCY peptide. (**c**,**d**): YCY peptide. (**e**,**f**): glutathione.

In this study, we synthesized fluorescent AuNCs by employing tyrosine-containing tripeptide as both a reducing agent and stabilizer (Scheme 1). Under alkaline conditions, the phenolate group in tyrosine was expected to reduce Au^3+^ to Au^0^ through electron transfer, resulting in the formation of a semi-quinone [29,40]. We increased the temperature to accelerate the reduction of Au^3+^ ions. The important condition in utilizing the reduction capability of tyrosine for the synthesis of AuNC is pH [19,41]. To optimize the pH condition for the synthesis of peptide-templated AuNCs, we added different amounts of 1 M NaOH to the mixture of HAuCl_4_ and the peptide. The purity of the synthesized peptides is shown in Figure S1 (Supplementary Materials). Glutathione was used as a control peptide as it has been used to synthesize fluorescent AuNC [32,42]. After an 8 h reaction at 70 °C, the strong fluorescent emission peak at 415 nm for both YCY- and SCY-AuNCs appeared with 325 nm excitation when the pH of the mixture was greater than 7 (Figure 1a,c). The strongest emission peak of YCY- and SCY-AuNCs was achieved at pH 10. Interestingly, an additional emission peak from YCY-AuNC was observed at 670 nm from 400 nm excitation (Figure 1d), but not from the SCY-AuNC. Under the acidic and neutral pH conditions, on the other hand, the GSH-AuNC gave fluorescence spectra with two distinct peaks at 390 nm and 800 nm from 325 nm excitation and with a peak at 800 nm from 400 nm excitation (Figure 1e,f). From this result, we found that the optimal pH condition for the synthesis of fluorescent AuNCs was pH 10, in which the tyrosine further facilitated the formation of AuNCs. 

Under the optimal condition, the color of the YCY solution changed from colorless to red, and the color of the SCY solution changed from colorless to brown during the reaction. The solutions of YCY- and SCY-AuNC were centrifuged to remove the sediment and agglomerated particles. A strong blue emission was observed under the 365 nm UV lamp in the supernatants of the YCY- and SCY-AuNC solutions. Interestingly, the precipitates of the centrifuged YCY-AuNC solution emitted deep-red fluorescence under the 365 nm UV lamp. We obtained red-emitting YCY-AuNCs from the precipitates using 10% acetic acid and DMSO (1:1 *v*/*v*). Afterward, we purified AuNCs by size exclusion chromatography to remove unreacted peptides, Au ions, and non-fluorescent gold nanoparticles, which were not removed by centrifugation (Figure S2, Supplementary Materials). After the dialysis, the AuNC solutions exhibited a neutral pH (~pH 7). 

### 2.2. The Optical and Physical Characteristics of the AuNCs

**Figure 2:**
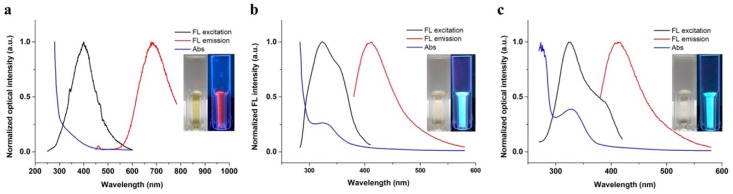
UV-Vis, fluorescent emission, and excitation spectra of (**a**) Red-YCY-AuNC, (**b**) Blue-YCY-AuNC, and (**c**) Blue-SCY-AuNC [photographs: AuNC solutions under visible light (left) and 365 nm UV lamp (right)].

Images in Figure 2 are Blue-YCY-AuNC, Blue-SCY-AuNC, and Red-YCY-AuNC under a 365 nm UV lamp showing uniform dispersity of the AuNCs in aqueous solution with a slight yellow color under the visible light. In the UV-vis spectra (Figure 2, blue curves), the absence of a surface plasmon resonance peak (520–530 nm) indicates that the synthesized AuNCs were smaller than 2.5 nm in diameter [43]. A distinct peak at 325 nm appeared in the UV-Vis absorption spectra of Blue-YCY-AuNC and Blue-SCY-AuNC. The strong blue emission wavelength (415 nm) of Blue-YCY-AuNC and Blue-SCY-AuNC was achieved when excited at 325 nm (Figure 2b,c). The emission peak of Red-YCY-AuNC appeared at 670 nm (Figure 2a). The fluorescence quantum yields of Red-YCY-AuNC, Blue-YCY-AuNC, and Blue-SCY-AuNC were 0.8%, 1.2%, and 1.5%, respectively.

**Figure 3:**
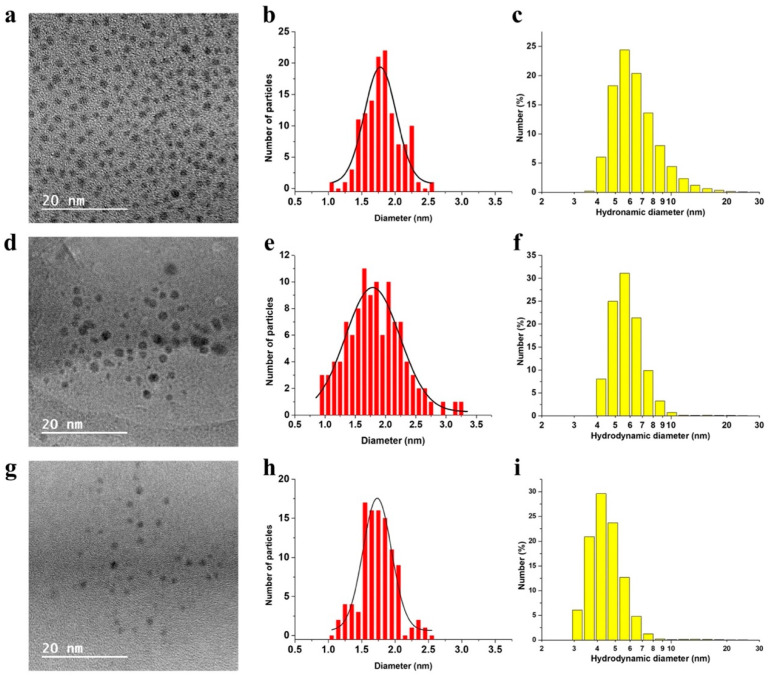
Red-YCY-AuNC: (**a**) TEM image, (**b**) size distribution histogram, and (**c**) DLS histogram. Blue-YCY-AuNC: (**d**) TEM image, (**e**) size distribution histogram, and (**f**) DLS histogram. Blue-SCY-AuNC: (**g**) TEM image, (**h**) size distribution histogram, and (**i**) DLS histogram.

High-resolution TEM images indicate that all the peptide-templated AuNCs were monodispersed with uniformity in size and spherical shape (Figure 3a,d,g). Size distribution histograms of each peptide-templated AuNC were constructed by measuring the diameters of 100 individual AuNCs (Figure 3b,e,h). The average size of the Blue-SCY-AuNC was found to be 1.7 ± 0.3 nm. Interestingly, Blue- and Red-YCY-AuNCs exhibited almost identical sizes of 1.8 ± 0.5 nm and 1.8 ± 0.3 nm, respectively. However, according to the DLS results, the hydrodynamic diameters of all peptide-templated AuNCs were larger than those obtained from TEM analysis (Figure 3c,f,i). This discrepancy can be explained by the external immobilization and the hydration of peptides on nanoclusters. Taken together, these experimental results demonstrate that we have successfully prepared fluorescent peptide-templated AuNCs without requiring any additional chemical-reducing agents.

**Figure 4:**
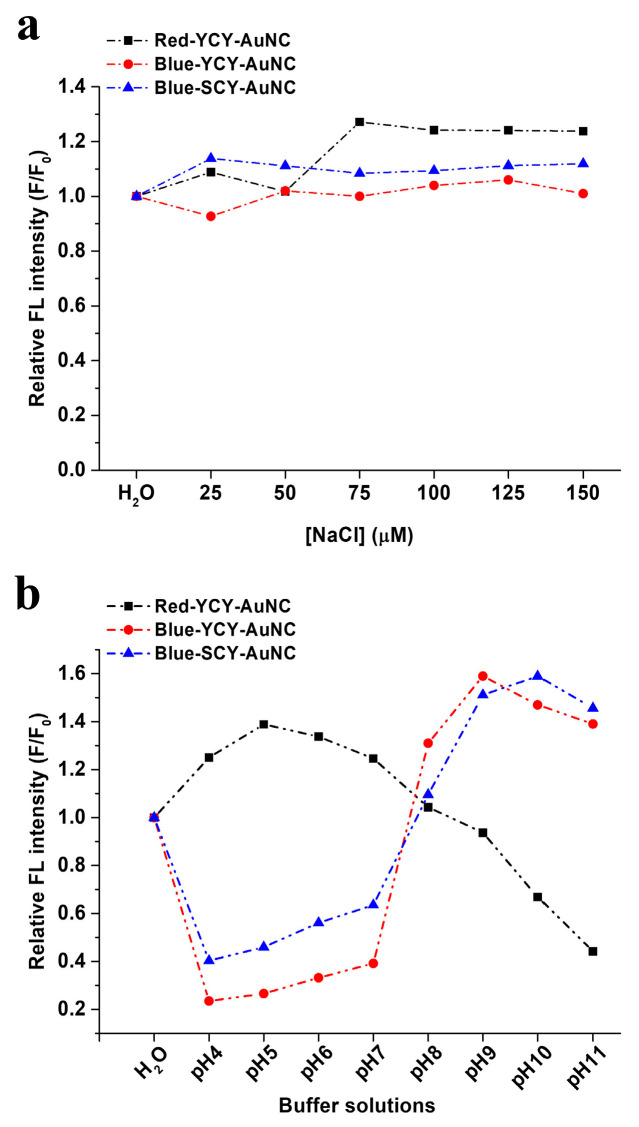
(**a**) Relative fluorescence intensity of Blue-YCY-AuNC, Blue-SCY-AuNC (λ_ex_: 325 nm, λ_em_: 415 nm), and Red-YCY-AuNC (λ_ex_: 400 nm, λ_em_: 675 nm) after mixing with various concentrations of NaCl (NaCl concentration shown on *x*-axis is after mixing). (**b**) Relative fluorescence intensity of Blue-YCY-AuNC, Blue-SCY-AuNC (λ_ex_: 325 nm, λ_em_: 415 nm), and Red-YCY-AuNC (λ_ex_: 400 nm, λ_em_: 675 nm) at different pH values.

As shown in Figure 4a, all AuNCs were stable under high ionic strength conditions. The stability was also maintained over time and at different temperatures (Figure S3, Supplementary Materials). To investigate the fluorescent response of the AuNCs to pH changes, we measured the fluorescence intensities of the AuNCs after mixing with buffer solutions with different pH values. As shown in Figure 4b (red circle and blue triangle,) the fluorescence intensities of Blue-YCY- and SCY-AuNCs decreased from pH 7 to pH 4. On the contrary, under the alkaline conditions, the fluorescence of the AuNCs showed an increase in the emission. Interestingly, Red-YCY-AuNCs exhibited the complete opposite trend of blue-emitting AuNCs (Figure 4b, black square). We speculate that the YCY and SCY peptides on the surface of Blue-YCY-AuNC and Blue-SCY-AuNC may have the same surface configurations to Au atoms, while the configuration of Red-YCY-AuNC is different. This notion can be supported by the results of the zeta potential measurement of each AuNC. Blue-YCY- and SCY-AuNCs exhibited −13.0 mV and −11.8 mV in H_2_O at the room temperature, while Red-YCY-AuNC exhibited +45.6 mV. It is possible that the YCY of Red-YCY-AuNC may adopt a configuration in which the O^−^ group of the unoxidized tyrosine and -SH of the cysteine form coordination bonds to Au atoms, resulting in a strongly positive charge in zeta potential for Red-YCY-AuNC [44]. In the case of blue-emitting AuNCs, the coordination bond only involves the -SH of the cysteine and the Au atoms, causing a weakly negative charge in blue fluorescence-emitting AuNCs.

### 2.3. Metal Selectivity and Sensitivity of AuNCs

**Figure 5:**
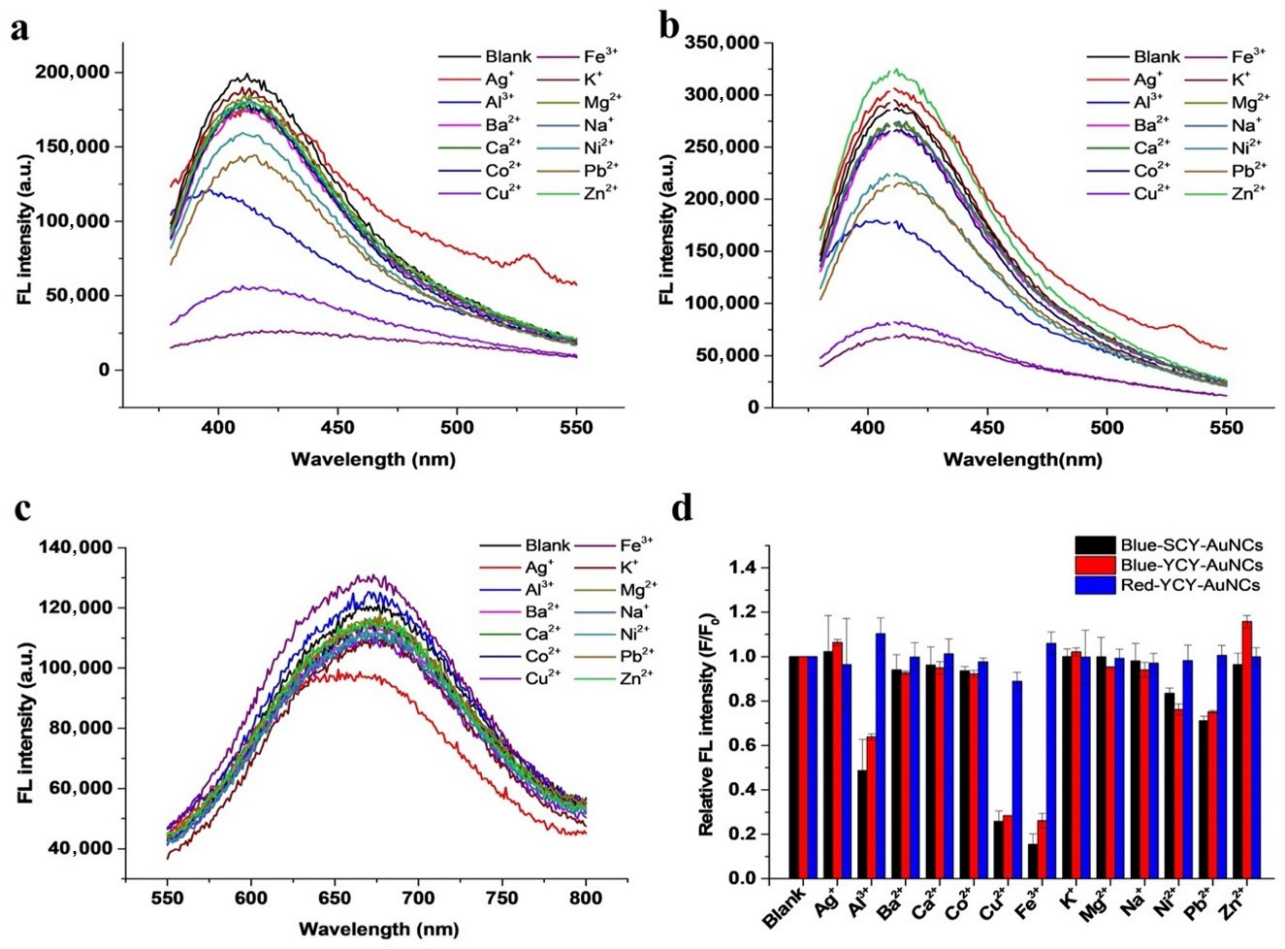
Fluorescence spectra of (**a**) Blue-YCY-AuNC, (**b**) Blue-SCY-AuNC, and (**c**) Red-YCY-AuNC after mixing with 13 different metal ions (50 μM, excitation at 325 nm and 400 nm for blue fluorescence-emitting AuNCs and red fluorescence-emitting AuNCs, respectively). Metal incubation time was 2 min. (**d**) Histogram plot of fluorescence quenching of each AuNC caused by 13 different metal ions.

**Figure 6:**
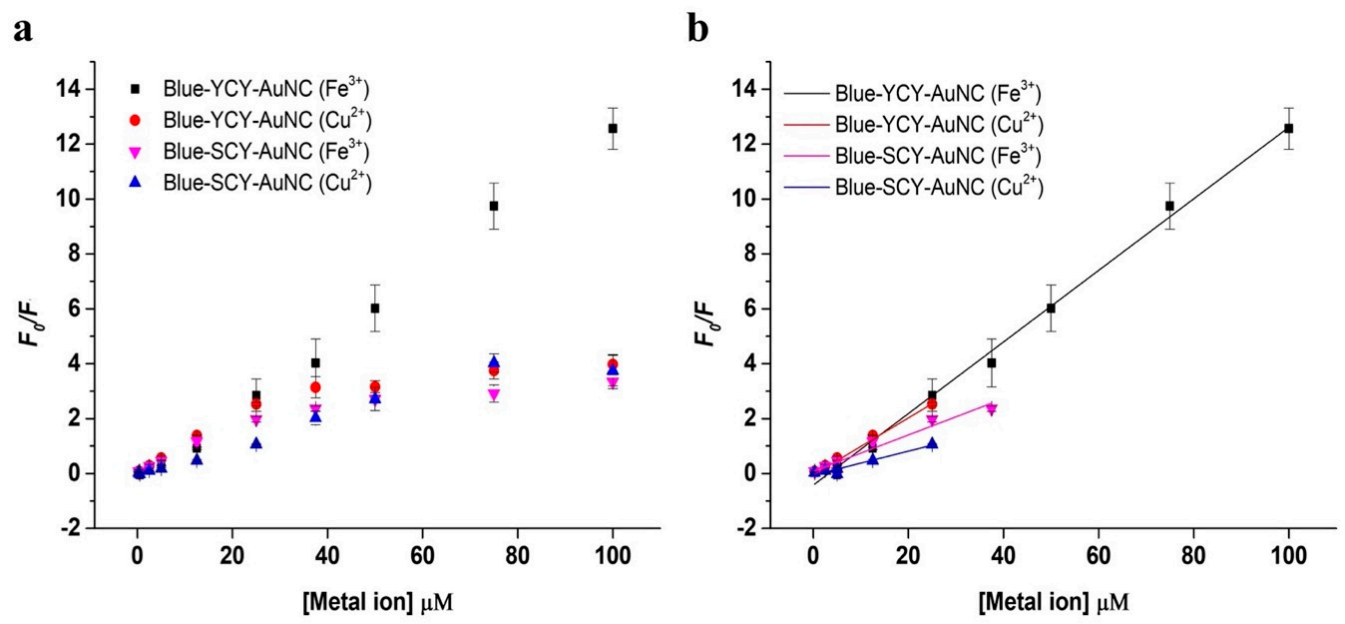
(**a**) Plot of F_0_/F at 415 nm versus the concentration of Fe^3+^ and Cu^2+^ (**b**) Stern–Volmer plot of Blue-YCY-AuNC and Blue-SCY-AuNC against Fe^3+^ (Blue-YCY-AuNC: 0.25–100 μM, Blue-SCY-AuNC: 0.25–37.5 μM) and Cu^2+^ (Blue-YCY-AuNC: 0.25–25 μM, Blue-SCY-AuNC: 0.25–25 μM). Excitation was at 325 nm. The error bars represent standard deviation from three independent measurements.

Tyrosine residue in a peptide is known to show a strong interaction with transition metal ions by forming a square planar complex in which the C-terminal carboxylate, amide nitrogen, and N-terminal amine bind to metal ions [44,45]. In addition, studies have suggested that the oxidized form of a tyrosine side chain, a semi-quinone, that is produced when Au^3+^ is reduced to Au^0^, may form complexes with Fe^3+^ and Cu^2+^ [44,45]. Therefore, we explored the sensing capability of the synthesized AuNCs to various metal ions. We mixed the same volume of the AuNC solution and 13 different metal ion solutions (100 μM) and measured fluorescence intensities. As shown in Figure 5a, significant fluorescence quenching (~85%) of Blue-YCY-AuNC was found in the presence of 50 μM Fe^3+^, and 75% fluorescence quenching of Blue-SCY-AuNC was observed compared to the blank sample (Figure 5b). Furthermore, Blue-YCY- and SCY-AuNCs responded to Cu^2+^ and Al^3+^ with ~75% and ~45% quenching efficiency, respectively (Figure 5a,b). On the other hand, Ag^+^, Ba^2+^, Ca^2+^, Co^2+^, K^+^, Mg^2+^, Na^+^, Ni^+^, Pb^2+^, and Zn^2+^ ions did not significantly affect the fluorescence signals of Blue-YCY- and SCY-AuNCs. The presence of other metal ions did not interfere with the fluorescence quenching of Blue-YCY- and SCY-AuNCs by these selected metal ions (Figure S4, Supplementary Materials). In addition, the fluorescence quenching of Blue-YCY- and SCY-AuNCs was found to coincide with the disappearance of a peak at 325 nm in UV-Vis absorption spectra (Figure S5, Supplementary Materials). Interestingly, the fluorescence signals of Red-YCY-AuNCs were stable for all 13 different metal ions (Figure 5d). Since both blue fluorescence-emitting AuNCs are more sensitive to Fe^3+^ and Cu^2+^, we further evaluated the interaction between blue fluorescence-emitting AuNCs and the two metal ions. We monitored changes in fluorescence under various concentrations of Fe^3+^ and Cu^2+^. The fluorescence-quenching data were fitted to the Stern–Volmer equation [46,47]:

*F*_0_*/F =* 1 + *K*_sv_[*Q*]

where *K*_sv_ is the Stern–Volmer quenching constant, [*Q*] is the analyte quenchers (Fe^3+^ or Cu^2+^), and *F*_0_ and *F* are the fluorescence intensities of the AuNCs at 415 nm in the absence and presence of metal ions. As observed in Figure 6a, the intensities of the fluorescence emission at 415 nm, in general, gradually decreased as the concentrations of Fe^3+^ and Cu^2+^ increased, up to approximately 40 μM when the emission began to plateau (summarized in Table 1). Blue-YCY-AuNC for Fe^3+^ sensing, on the other hand, did not exhibit this plateau trend. The fluorescence intensity continued to decrease with Fe^3+^ and Cu^2+^ concentrations, with a strong linear correlation R^2^ of 0.995 and 0.997 at concentrations ranging from 0.25 μM to 100 μM and from 0.25 μM to 25 μM, respectively (Figure 6b). In addition, the Blue-SCY-AuNC also had a strong linear relationship with Fe^3+^ (R^2^ = 0.965) and Cu^2+^ (R^2^ = 0.993) from 0.25 μM to 37.5 μM and from 0.25 μM to 25 μM, respectively. Furthermore, Blue-YCY-AuNC exhibited higher sensitivity to Fe^3+^ and Cu^2+^ than the Blue-SCY-AuNC (Table 1), possibly due to the additional tyrosine residue in Blue-YCY-AuNC. The limit of detection (LOD) was calculated from the slope of the plot of the Stern–Volmer equation versus concentrations of Fe^3+^ and Cu^2+^. The LOD value was calculated according to the assumption of an LOD equal to 3SD/S, where SD represents the standard deviation of the intercept and S represents the slope of the linear curve [32]. We obtained 3.2 μM and 0.77 μM LOD values for Blue-YCY-AuNC on sensing for Fe^3+^ and Cu^2+^, respectively. The LODs for Blue-SCY-AuNC were 4.1 μM and 1.3 μM for sensing Fe^3+^ and Cu^2+^. Therefore, the two-tyrosine containing peptide (YCY)-templated AuNC has better optical sensor ability toward to Fe^3+^ and Cu^2+^.

### 2.4. Sensing Mechanism

**Figure 7:**
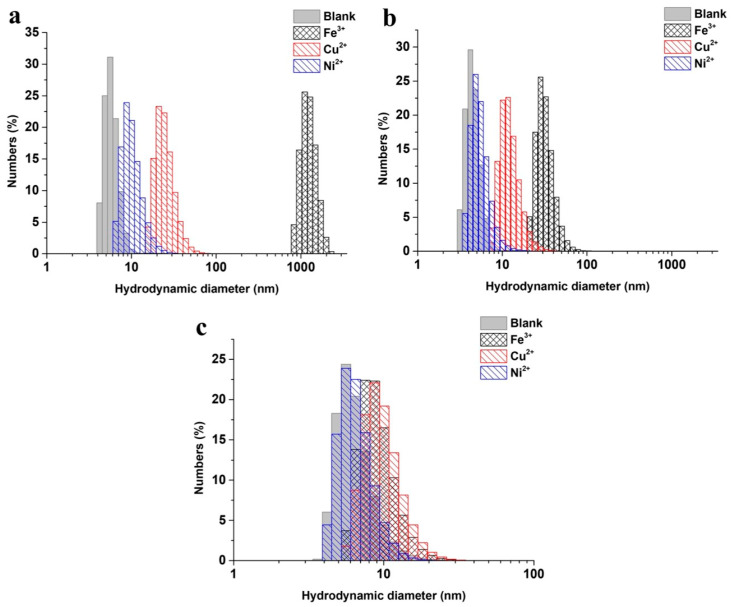
Analysis of hydrodynamic diameter of (**a**) Blue-YCY-AuNC, (**b**) Blue-SCY-AuNC, and (**c**) Red-YCY-AuNC before and after adding Ni^2+^, Fe^3+^, and Cu^2+^.

According to previous reports, ligands containing tyrosine or oxidized tyrosine can chelate metals such as Fe^3+^ and Cu^2+^ ions [44,48,49]. We examined the sensing mechanism by monitoring the hydrodynamic diameter of AuNCs after adding Fe^3+^ and Cu^2+^ to AuNC solutions. We also tested Ni^2+^ as a comparison to examine if the aggregation of AuNCs would occur in the presence of some specific target ions. The size-distribution histogram of each AuNC with Fe^3+^ and Cu^2+^ in Figure 7 revealed that the Blue-YCY-AuNC exceptionally tended to aggregate in the presence of Fe^3+^ ions, resulting in the formation of huge agglomerates (hydrodynamic diameter ~1200 nm). Similarly, when the Blue-YCY-AuNC was mixed with Cu^2+^, they formulated ~25 nm complexes, smaller than the complexes formed from the Blue-YCY-AuNC and Fe^3+^ (Figure 7a). The results strongly correlate with the tendency of the quenching efficiency of the Blue-YCY-AuNC toward Fe^3+^ and Cu^2+^ (Figure 5d). Under an identical circumstance, Fe^3+^ and Cu^2+^ induced the aggregation of Blue-SCY-AuNC, forming ~33 nm and ~12 nm complexes, respectively (Figure 7b). However, there was no significant aggregation in the presence of Ni^2+^ in both Blue-YCY- and SCY-AuNCs. In addition to these results, the DLS histogram of the Red-YCY-AuNC exhibited no aggregation even in the presence of Fe^3+^ and Cu^2+^ (Figure 7c). The results of DLS measurement have provided further evidence that the peptides on blue fluorescence-emitting AuNCs and Red-YCY-AuNC have different binding configurations to Au atoms. In the case of YCY on the Red-YCY-AuNC, as we proposed above, all the side chains’ functional groups were bound to the Au atom. Therefore, no functional groups were available to chelate to metal ions. In contrast, for YCY and SCY peptides on Blue-YCY- and SCY-AuNC, only the cysteine side chain -SH was bound to Au atoms. Therefore, the semi-quinone of the oxidized tyrosine and the N-terminal NH_2_ group can form complexes with Fe^3+^ and Cu^2+^, resulting in aggregation-induced fluorescence quenching [29,45,46]. These findings indicate that semi-quinones on the surface of AuNCs create aggregation by forming complexes with the other ligands of adjacent AuNCs. In addition, based on the DLS results, the aggregation propensity, and therefore the sensing capacity, of AuNCs strongly correlates with the number of possible semi-quinones on AuNCs.

## 3. Materials and Methods

### 3.1. Materials

*N*,*N*-Dimethylformamide (DMF), methanol (MeOH), acetonitrile (ACN), dimethyl sulfoxide (DMSO), ethyl ether, and dichloromethane (DCM) were purchased from Thermo Fisher Scientific (Waltham, MA, USA). Trifluoroacetic acid (TFA), 5,5′-dithiobis(2-nitrobenzoic acid) (DTNB), glutathione (GSH), *N,N*-diisopropylethylamine (DIPEA), and piperidine were obtained from Sigma-Aldrich (St. Louis, MO, USA). Nitrate salts of metal ions (AgNO_3_, Al(NO_3_)_3_, Ba(NO_3_)_2_, Ca(NO_3_)_2_, Co(NO_3_)_2_, Cu(NO_3_)_2_, Fe(NO_3_)_3_, KNO_3_, Mg(NO3)_2_, NaNO_3_, Ni(NO_3_)_2_, Pb(NO_3_)_2_, and Zn(NO_3_)_2_) were purchased from Sigma-Aldrich. Auric chloride (HAuCl_4_·3H_2_O) was obtained from Sigma-Aldrich. Fmoc-Tyr(*t*Bu)-OH, Fmoc-Cys(Trt)-OH, Fmoc-Ser(*t*Bu)-OH, Rink Amide MBHA resin (100–200 mesh), and 2-(1H-benzotriazol-1-yl)-1,1,3,3-tetramethyluronium hexfluorophosphate (HBTU) were purchased from Novabiochem (Darmstadt, Germany). 1-Hydroxybenzotriazole hydrate (HOBt·H_2_O) was obtained from Creosalus (Louisville, KY, USA). Sephadex^TM^ G-25 was purchased from GE Healthcare (Piscataway, NJ, USA). A dialysis membrane kit (Slide-A-Lyzer^®^Dialysis Cassette G2; 3.5 kDa cutoff) was obtained from Thermo Scientific (Waltham, MA, USA). Ultrapure water (18.2 MΩcm, Millipore Co. (Burlington, MA, USA)) was used in all experiments.

### 3.2. Synthesis of Tripeptides 

Tyrosine–cysteine–tyrosine (YCY) and serine–cysteine–tyrosine (SCY) peptides were synthesized using Rink Amide MBHA resins. First, 600 mg of Rink Amide MBHA resin was swelled in 3 mL of DMF for 1 h. Fmoc deprotection was achieved with 20% piperidine (20% in DMF *v*/*v*). After being washed 5 times with DMF, 2 times with MeOH, 2 times with DCM, and 3 times with DMF, a solution of 5 equivalents of Fmoc-Tyr(*t*Bu)-OH, HOBt·H_2_O, and HBTU, and 10 equivalents of DIPEA in dry DMF was added to the resins and mixed at room temperature for 3 h. All the resins were fully washed after each reaction step. The resins were treated with 20% piperidine for Fmoc deprotection and were mixed with a solution of Fmoc-Cys(Trt)-OH (5 eq.), HOBt·H_2_O (5 eq.), HBTU (5 eq.), and DIPEA (10 eq.) in dry DMF for 3 h. After Fmoc deprotection with 20% piperidine, the beads were divided equally into 2 fritted syringes. One syringe was mixed with a solution of Fmoc-Tyr(*t*Bu)-OH (5 eq.), HOBt·H_2_O (5 eq.), HBTU (5 eq.), and DIPEA (10 eq.) in dry DMF for 3 h. The other syringe was mixed with Fmoc-Ser(tBu)-OH (5 eq.), HOBt·H_2_O (5 eq.), HBTU (5 eq.), and DIPEA (10 eq.) in dry DMF. After the peptide-coupling reaction, the Fmoc-protecting group was removed with 20% piperidine. The peptides were cleaved and deprotected in a TFA cocktail solution (95% TFA, 2.5% TIS, 2.5% H_2_O) for 2 h and an additional 20 min with the fresh TFA cocktail solution. The solution was collected and evaporated under argon gas. When the volume of the solution was at 10% of the initial volume, chilled ethyl ether was added to the solution. The resulting solution was centrifuged at 4000× *g* and the supernatant was discarded. This step was repeated two times more. The precipitates were dried under vacuum. The resulting powders were dissolved in water with 0.1% TFA and purified by preparative RP-HPLC. Pure fractions were identified by HPLC-MS/MS (HPLC: Agilent, 1100 Series, MS/MS: Sciex, 4000 Q Trap). After purification, the purity of the tripeptides was evaluated by analytical HPLC-MS/MS. The combined pure fractions were lyophilized and kept at −20 °C.

### 3.3. Preparation of YCY- and SCY-Templated Fluorescent AuNCs

The concentration of the YCY- and SCY aqueous solutions was determined by Ellman’s reagent (DTNB). Glutathione was used as a standard reagent. The glass vial was cleaned using Aqua regia (HCl: HNO_3_, 3:1 *v*/*v*) and thoroughly rinsed with H_2_O prior to use. In the typical method for the synthesis of peptide-templated AuNCs, 0.5 mL of peptide solution (25 mM in H_2_O) was slowly added to 0.5 mL of HAuCl_4_∙3H_2_O (25 mM in H_2_O) with stirring. After mixing the solution for 15 min at 70 °C, the pH of the solution was adjusted to pH 10 with 1 M NaOH solution. The mixed solution was then continually stirred at 70 °C for 8 h. The solution was cooled to the room temperature, transferred to 1.5 mL individual microtubes, and centrifuged at 16,000× *g* for 10 min to remove the sediment and other impurities. The Blue-YCY-AuNCs and Blue-SCY-AuNCs were in the supernatant and the Red-YCY-AuNCs were trapped inside the precipitates after centrifugation. The Red-YCY-AuNCs were extracted by 1:1 of DMSO and 10% acetic acid aqueous solution. After 10 min of sonication, the solution was centrifugated at 16,000× *g* for 30 min to remove the precipitates. The yellow clear supernatants were transferred into new tubes. Each AuNC solution was purified via a Sephadex^TM^ G-25 column equilibrated and eluted with H_2_O (1 mL/min). Fractions of eluate (0.5 mL) were collected and analyzed by a UV-Vis absorption and fluorescence spectroscopy using a microplate reader. Fractions containing fluorescent AuNC were collected and dialyzed in H_2_O using a 3.5 kDa cutoff membrane to separate the AuNCs from any unreacted species and to adjust the pH to be neutral. The AuNC suspensions were stored at 4 °C in the dark for further characterization and experiment.

### 3.4. Characterization of AuNCs

UV-Vis spectra of the AuNC solution were measured with a microplate reader (Spark 20M; Tecan (Männedorf, Switzerland). Fluorescence spectra were obtained with a Horiba Fluorolog fluorometer (Horiba Scientific (Piscataway, NJ, USA)). The measurement was conducted with a 1 cm quartz cuvette at room temperature. The slits for the excitation and the emission were set to 5 nm. TEM images were taken using JEM-2100F (JEOL (Peabody, MA, USA)). A drop of the AuNC solution (0.1 mg/mL) was deposited on a carbon-coated Cu grid and analyzed. Dynamic light scattering (DLS) and zeta-potential were measured by Nano-ZS (Malvern Instrument (Westborough, MA, USA). 

### 3.5. The Influence of pH and NaCl on Fluorescence Intensity

The pH and the salt sensitivity of the AuNC were characterized by mixing 100 μL of each AuNC solution (Blue-YCY-AuNC and Blue-SCY-AuNC: 0.5 mg/mL, Red-YCY-AuNC: 0.15 mg/mL) with 400 μL of the different buffer solutions and NaCl solutions. The fluorescence emission spectra were obtained with 325 nm excitation and 400 nm excitation for blue fluorescence-emitting and red fluorescence-emitting AuNCs, respectively. 

### 3.6. Fluorescent Detections of Metal Ions

The following metal salts were used: Ag^+^, Al^3+^, Ba^2+^, Ca^2+^, Co^2+^, Cu^2+^, Fe^3+^, K^+^, Mg^2+^, Na^+^, Ni^2+^, Pb^2+^, and Zn^2+^. These metal salts were dissolved in H_2_O. Then, 300 μL of each AuNC solution (Blue-YCY-AuNC and Blue-SCY-AuNC: 0.2 mg/mL, Red-YCY-AuNC: 0.06 mg/mL) and 300 μL of each metal ion solution (100 μM) were mixed and the mixture was left to be equilibrated at room temperature for 2 min. Next, 500 μL of the solution was placed in the cuvette and the fluorescence-quenching spectra were measured from the appropriate excitation wavelength (Blue-YCY-AuNC and Blue-SCY-AuNC: 325 nm, Red-YCY-AuNC: 400 nm). Serial dilutions of Fe^3+^ and Cu^2+^ were also mixed with each AuNC solution and incubated for 2 min before the measurement.

### 3.7. Quantum Yield (QY) Measurement of AuNCs

Quantum yields of each AuNC were calculated using the following formula:

$$\Phi_{Χ}=\Phi_{ST}\left(\frac{{Grad}_{X}}{{Grad}_{\mathrm{ST}}}\right)\left(\frac{{\eta^{2}}_{X}}{{\eta^{2}}_{\mathrm{ST}}}\right)$$

where the subscripts ST and X denote standard and sample, respectively, Φ is the fluorescence quantum yield, Grad is the gradient from the plot of integrated fluorescence intensity versus absorbance, and η is the refractive index of the solvent. We used quinine sulfate (QY: 0.54) in 0.1 M H_2_SO_4_ (η: 1.33) as a fluorescence standard for the calculation of the QY of Blue-YCY- and Blue-SCY-AuNCs. For the measurement of the QY of Red-YCY-AuNC, 9,10-diphenyl anthracene (QY: 0.90) in cyclohexane (η: 1.44) was used as a reference fluorophore. We recorded the UV-vis absorbance spectrum of five serial solutions of each sample including a solvent background for the chosen references. Afterward, the fluorescence spectra of the same solution in the 1 cm fluorescence cuvette were measured. Graphs of integrated fluorescence intensity versus absorbance were plotted. 

## 4. Conclusions

In summary, by employing tyrosine-containing tripeptides, we successfully synthesized fluorescent AuNCs with emission wavelengths of 415 nm and 670 nm without using additional reducing agents. The as-prepared AuNCs displayed ultrasmall size (<2.5 nm), mono-dispersity, and uniform spherical shape in TEM images and DLS analyses. When the Blue-YCY-AuNC and Blue-SCY-AuNC were used to probe 13 metal ions, only Fe^3+^ and Cu^2+^ ions showed the significant fluorescence quenching of AuNCs. On the contrary, the Red-YCY-AuNC displayed a stable fluorescence signal in the presence of 13 metal ions. The Blue-YCY-AuNC showed higher sensitivity and a wider sensing range of Fe^3+^ and Cu^2+^ than the Blue-SCY-AuNC, with a lower detection limit of 3.2 μM and 0.77 μM, respectively. The interaction mechanism and sensing performance of AuNCs were systematically investigated using fluorescence spectra and DLS. In accordance with the fluorescence response of AuNCs to metal ions, Fe^3+^ induced the aggregation of the Blue-YCY-AuNC and formed larger complexes compared to the complexes formed with Cu^2+^. Furthermore, the Blue-SCY-AuNC induced aggregation in the presence of Fe^3+^ and Cu^2+^, though these complexes were smaller than those of the Blue-YCY-AuNC. Overall, although the number of tyrosine residues may not have a significant impact on the formation of AuNCs, the number of oxidized tyrosines (semi-quinone form) on the surface of AuNCs may play a critical role in the detection of target metal ions. This study provides a new and facile strategy to design surface ligands for AuNCs to sense metal ions.

In future work, we plan to integrate density functional theory calculations and molecular dynamics simulations to deepen our understanding of molecular interactions within synthesized gold nanoclusters. We also aim to expand our characterization efforts, particularly through mass spectrometry and other analytical methods, to accurately determine the size, charge, and ligand numbers for all nanoclusters.

## Figures and Tables

**Table 1:** Comparison of Blue-YCY-AuNC and Blue-SCY-AuNC for sensing Fe^3+^ and Cu^2+^.

Sample	Detection Metal Ion	Slope & Intercept	R^2^	Linear Detection Range (μM)	Limit of Detection (μM)
Blue-YCY-AuNC	Fe^3+^	Y = 0.128x − 0.314	0.995	0.25–100	3.2
	Cu^2+^	Y = 0.101x + 0.0533	0.997	0.25–25	0.77
Blue-SCY-AuNC	Fe^3+^	Y = 0.0646x + 0.144	0.965	0.25–37.5	4.1
	Cu^2+^	Y = 0.0424x − 0.0144	0.993	0.25–25	1.3

## Data Availability

The data that support the findings of this study are available on request from the corresponding author.

## Supplementary Materials

The following supporting information can be downloaded at https://www.mdpi.com/article/10.3390/molecules29112416/s1: Figure S1. HPLC-MS/MS of (a) YCY and (b) SCY; Figure S2. UV-Vis absorption and fluorescence intensity of the fractions from the size exclusion chromatography (Sephadex^TM^ G-25); Figure S3. Fluorescence intensity of the AuNC solutions over time at 4 °C, 25 °C, and 37 °C; Figure S4. Fluorescence quenching of Blue-SCY-AuNC (a) and Blue-YCY-AuNC (b) by Cu^2+^ (50 μM) and Fe^3+^ (50 μM) in the presence of K^+^ (50 μM) and Mg^2+^ (50 μM); Figure S5. UV-Vis absorption spectra of Blue-SCY-AuNC (a) and Blue-YCY-AuNC (b) in the presence or absence of Cu^2+^ ions (50 μM).
