# Supplementary material for: Tripeptide-Assisted Gold Nanocluster Formation for Fe3+ and Cu2+ Sensing
Source: Molecules. 2024 May 21;29(11):2416. doi: 10.3390/molecules29112416 (PMC11173388; doi:10.3390/molecules29112416)
Supplement: Supplementary file 1 [file molecules-29-02416-s001.zip › molecules-2985124-supplementary.pdf]

Supplementary Materials

Tripeptide-Assisted Gold Nanocluster Formation for Fe^{3+} and Cu^{2+} Sensing

Jonghae Youn [1,2], Peiyuan Kang [2], Justin Crowe [3], Caleb Thornsbury [3], Peter Kim [1], Zhenpeng Qin [2,4,5,6] and Jiyong Lee [3,*]

[1] Department of Chemistry and Biochemistry, The University of Texas at Dallas, Richardson, TX 75080, USA
[2] Department of Mechanical Engineering, The University of Texas at Dallas, Richardson, TX 75080, USA
[3] Department of Chemistry and Biochemistry, The University of Texas at Tyler, Tyler, TX 75799, USA
[4] Department of Bioengineering, The University of Texas at Dallas, Richardson, TX 75080, USA
[5] Department of Surgery, The University of Texas Southwestern Medical Center, Dallas, TX 75390, USA
[6] Center for Advanced Pain Studies, The University of Texas at Dallas, Richardson, TX 75080, USA
* Correspondence: jiyonglee@uttyler.edu

a

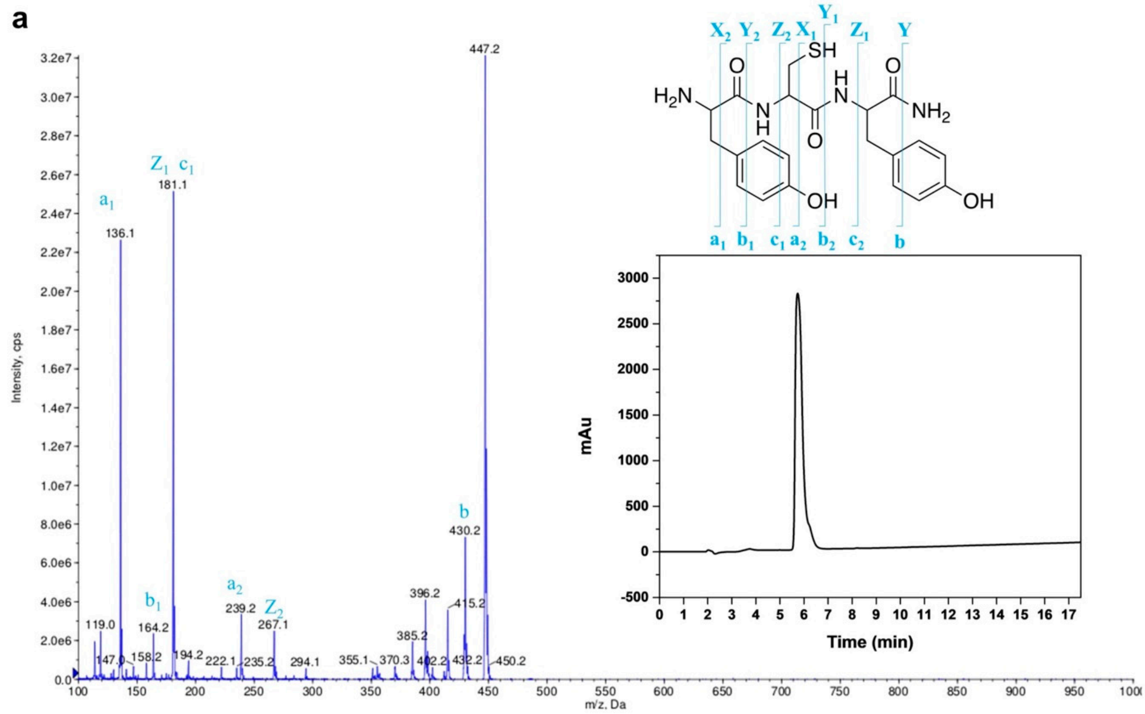

b

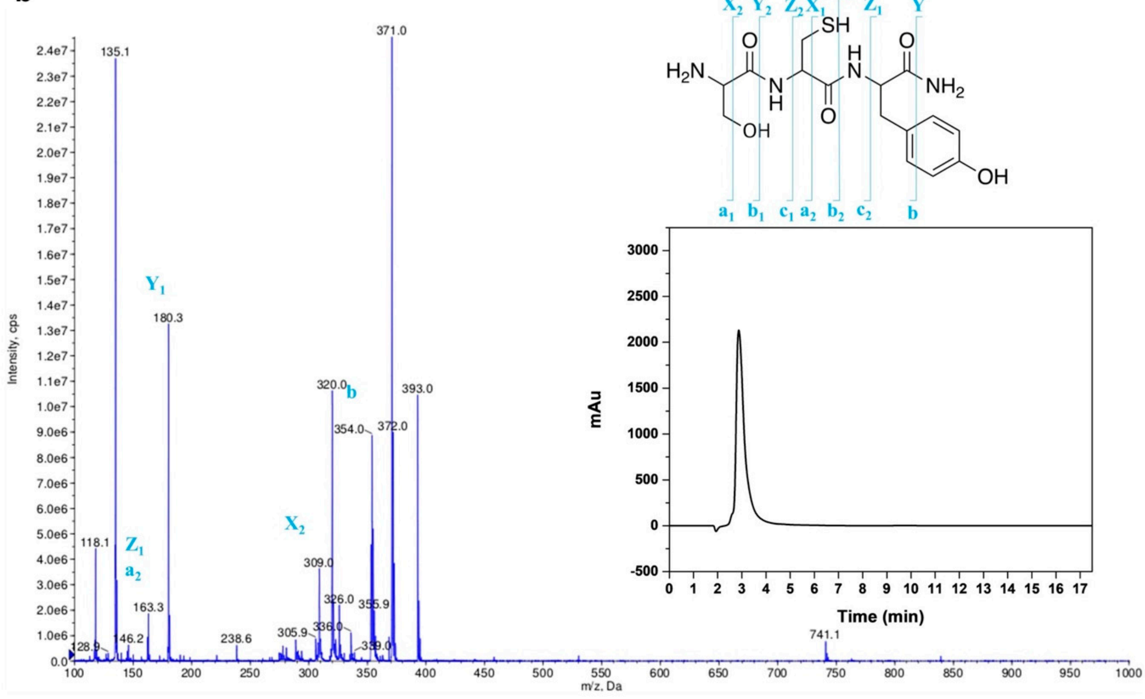

Figure S1. HPLC-MS/MS of (a) YCY and (b) SCY. Insets show analytical HPLC chromatogram of each peptide. LC gradient for YCY: 0% to 30% acetonitrile over 20 min, LC gradient for SCY: 0% acetonitrile over 20 min.

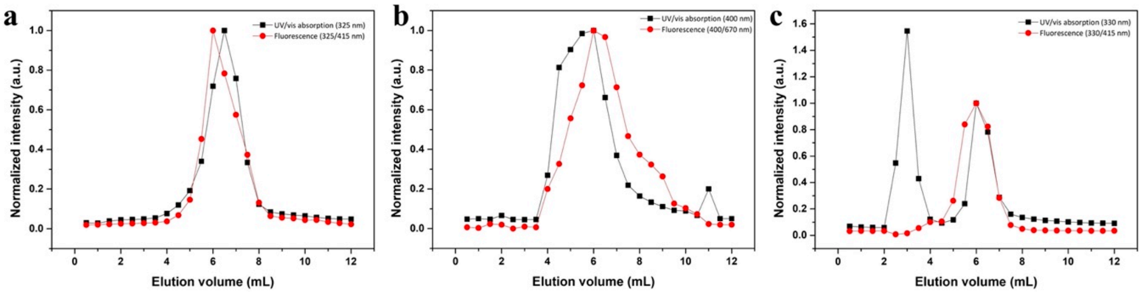

Figure S2. UV-Vis absorption and fluorescence intensity of the fractions from the size exclusion chromatography (Sephadex^{TM} G-25): (a) Red-YCY-AuNC, (b) Blue-YCY-AuNC, and (c) Blue-SCY-AuNC.

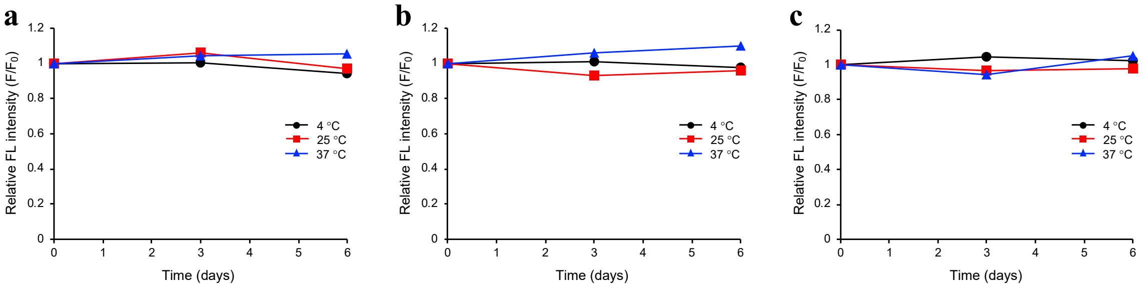

Figure S3. Fluorescence intensity of the AuNC solutions over time at 4 °C, 25 °C and 37 °C: (a) Blue-SCY-AuNC (λ_{ex}: 325 nm, λ_{em}: 415 nm), (b) Blue-YCY-AuNC (λ_{ex}: 325 nm, λ_{em}: 415 nm), and (c) Red-YCY-AuNC (λ_{ex}: 400 nm, λ_{em}: 675 nm).

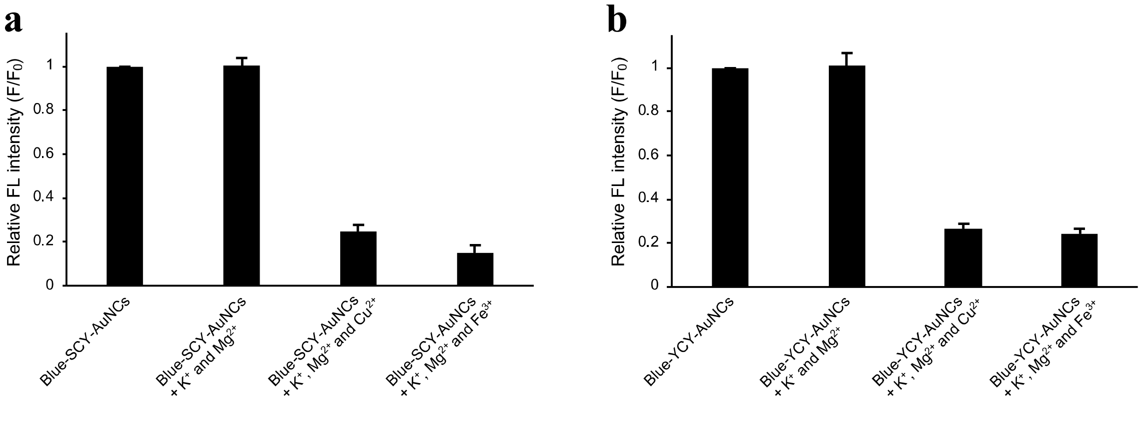

Figure S4. Fluorescence quenching of Blue-SCY-AuNC (a) and Blue-YCY-AuNC (b) by Cu^{2+}(50 µM) and Fe^{3+} (50 µM) in the presence of K^{+} (50 µM) and Mg^{2+} (50 µM). Fluorescent emission at 415 nm was measured with 325 nm excitation.

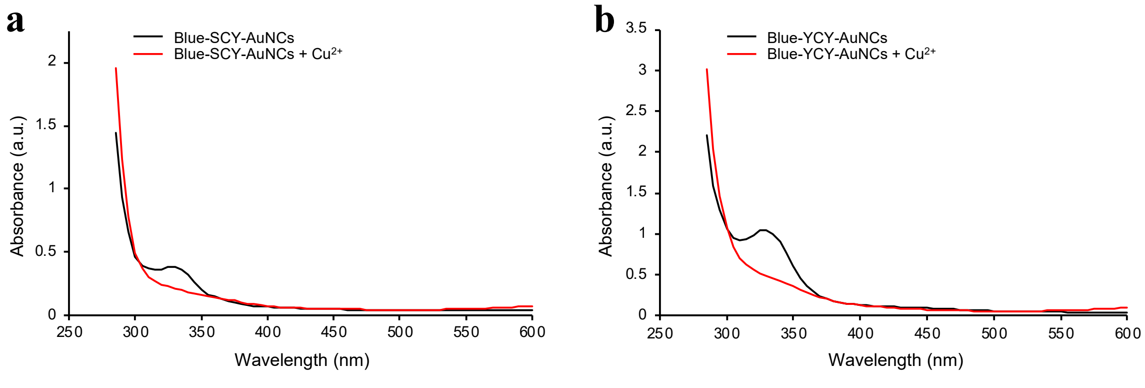

Figure S5. UV-Vis absorption spectra of Blue-SCY-AuNC (a) and Blue-YCY-AuNC (b) in the presence or absence of Cu^{2+} ions (50 µM). The effect Fe^{3+} ions on UV-Vis spectrum was not examined due to the spectral overlap of Fe^{3+} ions and the AuNCs.
